# Development of a CO_2_ Sensor for Extracorporeal Life Support Applications

**DOI:** 10.3390/s20133613

**Published:** 2020-06-27

**Authors:** Michele Bellancini, Laura Cercenelli, Stefano Severi, Guido Comai, Emanuela Marcelli

**Affiliations:** 1 Department of Electrical, Electronic and Information Engineering “Guglielmo Marconi” (DEI), Alma Mater Studiorum University of Bologna, 40136 Bologna, Italy; michele.bellancini2@unibo.it (M.B.); stefano.severi@unibo.it (S.S.); 2 MediCon Ingegneria s.r.l, 40054 Budrio, Italy; guido.comai@mediconingegneria.it; 3 Laboratory of Bioengineering, Department of Experimental Diagnostic and Specialty Medicine (DIMES), Alma Mater Studiorum University of Bologna, 40138 Bologna, Italy; emanuela.marcelli@unibo.it

**Keywords:** CO_2_ sensor, mid-IR, extracorporeal life support devices

## Abstract

Measurement of carbon dioxide (CO_2_) in medical applications is a well-established method for monitoring patient’s pulmonary function in a noninvasive way widely used in emergency, intensive care, and during anesthesia. Even in extracorporeal-life support applications, such as Extracorporeal Carbon Dioxide Removal (ECCO_2_R), Extracorporeal Membrane Oxygenation (ECMO), and cardiopulmonary by-pass (CPB), measurement of the CO_2_ concentration in the membrane oxygenator exhaust gas is proven to be useful to evaluate the treatment progress as well as the performance of the membrane oxygenator. In this paper, we present a new optical sensor specifically designed for the measurement of CO_2_ concentration in oxygenator exhaust gas. Further, the developed sensor allows measurement of the gas flow applied to the membrane oxygenator as well as the estimation of the CO_2_ removal rate. A heating module is implemented within the sensor to avoid water vapor condensation. Effects of temperature on the sensor optical elements of the sensors are disclosed, as well as a method to avoid signal–temperature dependency. The newly developed sensor has been tested and compared against a reference device routinely used in clinical practice in both laboratory and in vivo conditions. Results show that sensor accuracy fulfills the requirements of the ISO standard, and that is suitable for clinical applications.

## 1. Introduction

Capnometry is the measurement of carbon dioxide (CO_2_) concentration in respiratory gases [1]. It is a well-known and established method for monitoring patient’s pulmonary function in a noninvasive way, widely used in emergency situations, as well as in intensive care or during anesthesia [2,3,4,5]. Even if the traditional use of capnometry is related to the field of respiratory monitoring, the application of this measurement in extracorporeal life support (ECLS) systems such as Cardio-Pulmonary Bypass (CPB) [6], Extracorporeal Membrane Oxygenation (ECMO) [7], and Extracorporeal Carbon Dioxide Removal (ECCO_2_R) [8] has been proposed. The goal of these procedures is to add oxygen (O_2_) and remove carbon dioxide (CO_2_) from patient blood, which is pumped through a membrane oxygenator (MO) where, gas exchange between the blood and sweep gas in the MO takes place. Therefore, the measurement of CO_2_ removed by the MO can be achieved placing a capnometer (CO_2_ sensor) at the exhaust port of the MO. This method is called oxygenator exhaust capnometry. All the procedures mentioned above represent complex systems that require strict monitoring of both patient and device conditions. In particular, monitoring of MO performance is crucial during extracorporeal circulation procedures, as it might indicate a functional impairment due to clot formation within the MO and therefore the need for MO replacement. In this context, the analysis of CO_2_ concentration in the MO exhaust can be exploited to evaluate the so-called dead space, i.e., the portion of the MO that is ventilated, but not perfused by blood, and therefore not participating in gas exchange [7]. During ECMO procedure, monitoring of MO performance may provide information regarding both the patient’s lung status and the MO contribution to the global ventilation, therefore guiding the weaning process [9]. In CPB procedures, the oxygenator exhaust capnometry has been proven to be a useful tool for continuously estimating the CO_2_ concentration in arterial blood (P_a_CO_2_) in a noninvasive way, representing an alternative to gas blood analysis [10]. Further, variation in P_a_CO_2_ during ECMO procedures is proven to be associated with higher mortality, due to the dangerous effect of high CO_2_ level to brain circulation [11]. In ECCO_2_R, which is a procedure for treatment of hypercapnia (i.e., a condition of abnormally elevated CO_2_ level in the blood) [12], the measurement of CO_2_ concentration in the exhaust gas and of CO_2_ removal rate represents a key parameter to estimate the treatment progress. Therefore, CO_2_ monitoring in the exauhst gas of a MO is a useful tool for monitoring patient’s status and MO conditions as well as for evaluating the progress of the extracorporeal procedure. In the ECLS context, a flow sensor can be used to gather information of sweep gas flow (GF). Combining the information about GF and CO_2_ concentration, the removal rate of CO_2_ (VCO_2_) expressed as [mL/min] can be obtained, this value represents the volume of CO_2_ removed over time. Further, measurement of GF can be used to detect a failure or a disconnection of the gas flow line in the MO. Despite its relative ease, the oxygenator exhaust capnometry is not routinely applied, as some practical issues remain unsolved in providing a reliable measurement of CO_2_ removal from the patient. Even though several CO_2_ sensors for medical applications are available, such as Masimo AB EMMA™ [13] and Medtronic Microcap™ [14], these are intended for respiratory monitoring and are not suitable for direct use in ECLS procedures. One of the main obstacles to CO_2_ measurement at the exhaust port of a MO is the condensation of the water vapor contained in the gas exhaust on the optical elements of the CO_2_ sensor. The water vapor condensation causes degradation of the signal acquired by the sensor and therefore an incorrect estimation of CO_2_ concentration. This paper presents a newly developed sensing platform designed explicitly for ECLS application comprising a CO_2_ sensor and a flow sensor. A solution to avoid condensation of the water vapor on the CO_2_ sensing elements is described, as well as the accuracy of the CO_2_ measurements provided by the new sensor in comparison with commercial devices and reference standard requirements.

## 2. Materials and Methods

### 2.1. State-of-the-Art on CO_2_ Sensors

**Figure 1:**
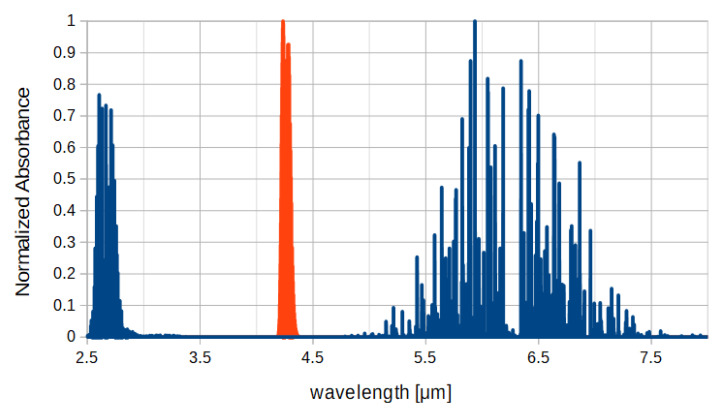
Absorption bands of CO_2_ (orange) and H_2_O (blue) [19].

Optical-based sensors represent the state-of-the-art in the measurement of CO_2_ concentration in gases [15]. These are typically based on infrared spectroscopy, a well-known technique that exploits the ability of molecules to absorb light at specific wavelengths related to vibration and rotation mechanisms of molecules [16]. The Bourger–Lambert–Beer Law describes the working principle of these sensors [17]:

(1) $$dI=-\alpha \left(\lambda \right)cI_{o}dx$$

where *I* is the transmitted radiation intensity, Io is the incident radiation intensity, *c* is the analyte concentration, α(λ) is the analyte-specific absorption coefficient, and *x* represent the optical path length. The integral form of the Bourger–Lambert–Beer’s Law,

(2) $$I=I_{o}e^{-\alpha (\lambda )cx}$$

shows that it is possible to calculate the analyte concentration observing variation of the transmitted radiation *I*, as it represents attenuation of the incident radiation *Io* once it has passed through the optical path *x* containing an amount *c* of the analyte with absorption coefficient α(λ). In Equation (2) is highlighted that the absorption coefficient is a wavelength-dependent parameter, so to correctly exploit the absorption measurement principle is of paramount importance that the incident light has a spectrum that includes the absorption band of the analyte of interest.For CO_2_, the incident light spectrum shall be in the mid-infrared (mid-IR) region (3–8 μm) as the principal absorption peak for carbon dioxide is located at 4.25 μm [18]. Further, to obtain a direct measurement of the analyte of interest, no other compounds shall have absorption bands included on the incident light spectrum. Therefore, the whole composition of the oxygenator exhaust gas mixture has to be considered in order to develop an efficient CO_2_ sensor for ECLS applications. The exhaust gas of a membrane oxygenator during ECLS procedure contains a variable concentration of the following chemical species: Oxygen (O_2_), Nitrogen (N_2_), Carbon Dioxide (CO_2_), and water vapor. As infrared radiations are absorbed only by asymmetrical molecules, only CO_2_ and water vapor (H_2_O) have absorption bands in the mid-IR region. Absorption spectra of CO_2_ and H_2_O obtained from the HITRAN database [19] are reported in Figure 1.

**Figure 2:**
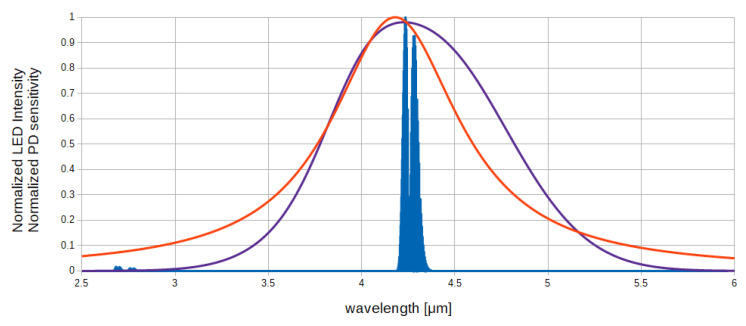
InAsSb/InAs LED (red) and PD (purple) spectra. CO_2_ absorption band in the 4.2 μm region (blue) [19].

Methods to obtain emission in the mid-IR spectrum used in the currently available capnometers (CO_2_ sensors) involve the use of a broadband emitting incandescence light source coupled with thermopiles, pyroelectric detectors, or infrared photodiodes [20]. Use of a broadband emitting light source impose to add to the system a solution to filter the unwanted radiation, such as a beam splitter, optical filters, and a rotating filter wheel [21]. An alternative consists in the use of a narrow spectrum emitting diode coupled with an appropriate photodetector. This solution is possible thanks to the use of light-emitting diode (LED) and photodetector (PD) realized with InAsSb epitaxial layer on InAs substrate (InAsSb/InAs) [22]. Optical coupling of InAsSb/InAs LED and PD allows the emission and detection of IR radiation near 4.2 μm that comprises the principal CO_2_ absorption peak, avoiding the use of mechanical modulators and interference filters and therefore allowing a simpler design of the sensor. Further, working in the region near 4.2 μm allows the direct measurement of CO_2_ even in the presence of water vapor. In Figure 2, emission and detection spectra are shown. Note that, as LED and PD spectra overlap the CO_2_ absorption band, the measurement of CO_2_ concentration is possible.

In commercially available optical CO_2_ sensors for medical applications, two different approaches are used for measurement of CO_2_ concentration: Main-stream and Side-stream [23]. In Main Stream sensors the measurement is taken on the main gas flow, using a measuring chamber that allows the emitted optical beam to cross the gas and to reach the detector, whereas in Side Stream sensors a portion of the gas flow is pulled by the sensors into a measurement chamber where the optical elements are positioned.

### 2.2. Design Requirements for CO_2_ Sensors Applied to ECLS Procedures

Regarding the CO_2_ sensor requirements intended for ECLS procedures, the following aspects shall be considered. First, a certain accuracy level is required for CO_2_ measurement. Minimum accuracy for a CO_2_ sensor in the medical field is defined by the standard ISO 80601-2-55 [24] as ±0.43% + 8% of the CO_2_ concentration. A second aspect to take into account is that in ECLS procedures, the water vapor condensation at the exhaust port of the MO occurs, therefore the CO_2_ sensor should implement a method to avoid the water vapor condensation on optical elements; otherwise, the optical signal deterioration due to condensation will lead to incorrect estimation of CO_2_ concentration. Some capnometers for the measurement of CO_2_ concentration in the exhaled breath, use a heating system to increase the temperature in the measuring chamber, avoiding water vapor condensation [25]. It is noteworthy that the effect of water vapor condensation is worse in ECLS procedures than in exhaled breath monitoring, due to the presence of a continuous gas flow and higher flow rate at the oxygenator exhaust port. Therefore, considering the water vapor condensation effect, in ECLS procedure a Main-stream CO_2_ sensor is more appropriate than a Side-stream sensor, as in the latter, the water vapor can easily condensate in the tube used to bring the gas sample to the measuring chamber, thus leading to clotting of the sample line and compromising the correct measurement [26]. Further, the use of Side-stream CO_2_ sensor at the oxygenator exhaust port has the drawback of the environmental air pulled to the measuring chamber together with the exhaust gas, which leads to the incorrect estimation of CO_2_ removed from the patient [27]. An answer to this problem may consist in placement of the sampling tube inside the MO exhaust port [28]. This solution brings to sterilization issues that are not compatible with clinical routine in intensive care unit (ICU). An additional requirement that a CO_2_ sensor for ECLS application should fulfill is the possibility to get the carbon dioxide removal rate VCO_2_. Therefore, a method to measure the gas flow (GF) applied to the MO should be implemented. Regarding time resolution of the obtained measurement, differently form respiratory capnometry in which high time resolution is necessary to obtain information on the end-tidal CO_2_ and respiratory rate, capnometry applied to ECLS procedures, does not require such highly time-resolved measurement, as in ECLS applications the oxygenator exhaust gas flow and CO_2_ concentration change slowly. Therefore, temporal resolution of the order of minutes is sufficient to extract information about the ECLS procedure, that are used for evaluation of long-term therapy progress and trends. Finally, a CO_2_ sensor for ECLS application should not be designed as a standalone device, but rather it should be interfaced with monitoring devices already used to monitor the patient’s condition or to the ECLS device itself.

### 2.3. The Newly Developed CO_2_ Sensing Platform

**Figure 3:**
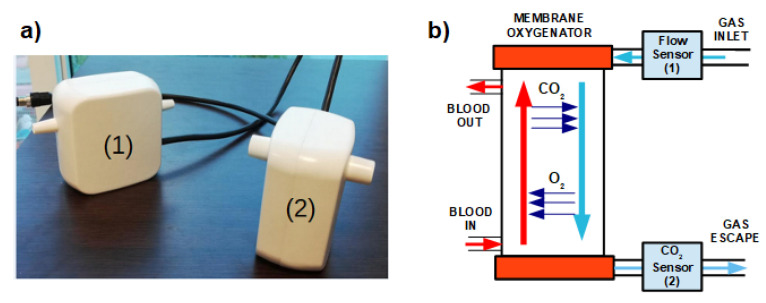
(**a**) Developed CO_2_ sensing platform. (1) Flow measurement section; (2) CO_2_ measurement section. (**b**) Schematic representation of flow sensor and CO_2_ sensor on MO.

The proposed solution consists of a sensing platform made up of two sections: one for the GF measurement and the other one for CO_2_ measurement, as illustrated in Figure 3. The flow measurement section contains an off-the-shelf mass flow sensor (SFM4100, Sensirion AG, Staefa, Switzerland) able to measure GF up to 20 SLM (Standard Litre per Minute) with accuracy of 3% on the measured value. As stated before, measurements taken at MO exhaust port are affected by water vapor condensation. Differently from the CO_2_ sensor, increment of the sensor temperature is not a suitable solution to avoid water vapor condensation in the GF measurement section, as it would interfere with the measurement principle exploited by the flow sensor, that depends on the cooling effect applied by a gas flow on a heating element placed within the sensor [29]. Therefore, to avoid measurement error due to water vapor, the GF measurement section has been designed to be positioned at the inlet port of the MO. Further, measurement of GF at the inlet port of the MO provides to the operator a direct feedback about the sweep gas flow used for the extracorporeal procedure. Data collected by the flow sensor are communicated through I2C interface to the CPU contained in the CO_2_ measurement section.

The CO_2_ measurement section has been entirely developed and is made up by three subsections:

1. emission stage for the generation of the mid-IR beam;
2. receiver stage for the detection, conditioning and amplification of the optical signal after CO_2_ absorption and;
3. CPU for signal acquisition, processing, and communication with a host device.

**Figure 4:**
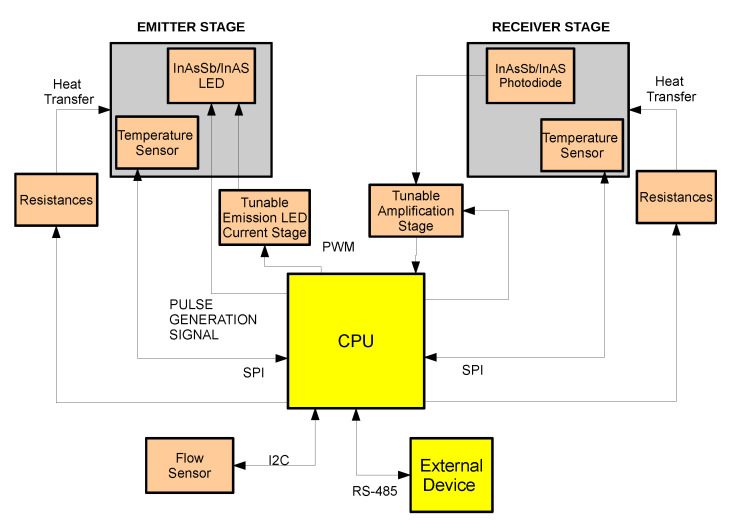
Schematic representation of the sensor.

**Figure 5:**
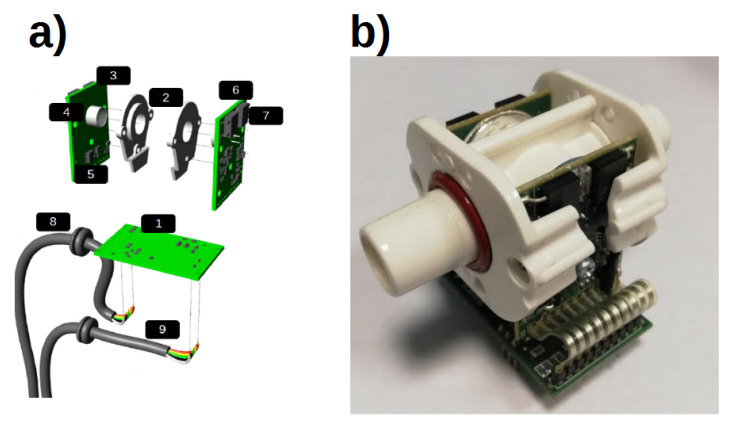
(**a**) Exploded drawing of the CO_2_ sensor: (1) CPU board; (2) aluminum rings; (3) emitter board; (4) InAsSb/InAs element; (5) digital temperature sensor; (6) receiver board; (7) heating resistances; (8) Flow sensor communication cable; (9) power supply/RS-485 cable. (**b**) Assembly of the developed CO_2_ and plastic cuvette.

For both emission and detection of the mid-IR beam, optical elements of InAsSb/InAs (LED Microsensor NT, Saint-Petersburg, Russia) are used [30]. A parabolic reflector is mounted on both LED and PD to improve directivity of the mid-IR beam and therefore the optical coupling. LED and PD are placed on opposite sides of a measuring cuvette, and are mechanically fixed in order to assure the correct positioning and optimal optical coupling. To protect the optical elements, a sapphire glass window is mounted on the parabolic reflector. As only a single LED-PD couple is used, this solution represents a single-channel architecture. Use of the single-channel architecture allows to obtain a simpler device, easy to assemble and cheaper, as no optical filters or beam splitter are used. As InAsSb/InAs elements are affected by the variability in their optical performance in terms of emission efficiency and photosensitivity, the microcontroller can modulate the LED current and amplification gain of the receiver stage allowing the proper tuning of each optical couple in order to achieve the best performance. Measuring chamber of the sensor has been designed to be connected directly to the MO exhaust port connector, therefore implementing a Main Stream architecture since the sample gas flows directly in the measuring chamber of the developed CO_2_ sensor. A Heating system is implemented both on emitter and receiver stage, in order to avoid water vapor condensation. The Heating system is composed of resistances mounted on rear of the electronics boards that generates heat, an aluminum ring that surrounds the optical elements and conduce the generated heat along the measuring chamber, and a digital temperature sensor for the temperature monitoring. CPU controls the temperature of the emitter and the receiver stages by switching ON and OFF the current circulating in the resistances. A block diagram of the sensor and a drawing of the CO_2_ section are reported in Figure 4 and Figure 5.

**Figure 6:**
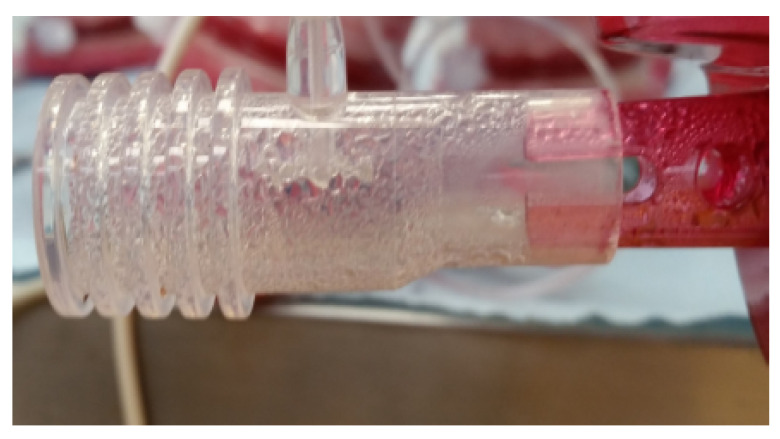
Effect of water vapor condensation on a test cuvette placed at the exhaust port of a membrane oxygenator.

Thanks to the implemented solution, the water vapor condensation is prevented not only directly on optical elements but also in their proximity, obtaining a water-free cuvette. Figure 6 shows an example of water vapor condensation on a cuvette placed at MO exhaust port that occurs without the implementation of a heating system, leading to a complete degradation of the optical signal.

**Figure 7:**
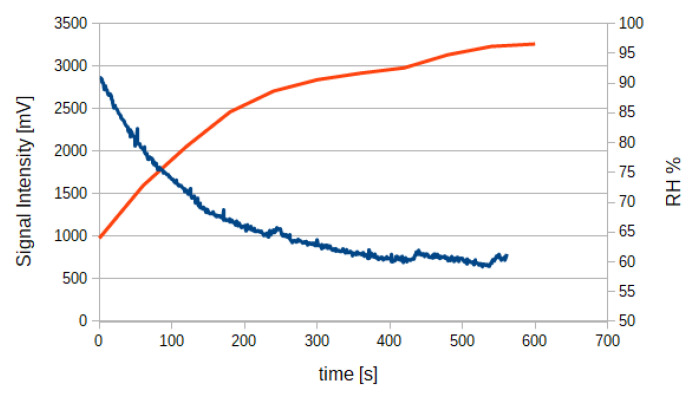
Optical signal intensity (blue) versus relative humidity (orange).

Further, in Figure 7 is reported the experimental result obtained recording optical signal from CO_2_ sensors without the implemented heating system while relative humidity (RH%) of the gas flow increases. As RH% increases, water vapor starts to condensate on the measuring chamber walls and on optical elements, leading to signal degradation.

Gas temperature at the exhaust port of a MO usually is 38 °C during ECLS procedures such as CPB [31]; therefore, increasing the temperature of the sensor measuring chamber to 40 °C prevents water vapor condensation. Even though the heating of the sensor’s measuring chamber prevents water vapor condensation, an increase in optical elements temperature brings side effects relevant to LED and PD efficiency. Moreover, the goal of the implemented heating system is to prevent water vapor condensation not only on optical elements but also on the entire measuring chamber, preventing formation of water drops that over time will move on the optical elements. For this reason, heating elements larger than the optical elements package has been used. This solution allows a more efficient diffusion of heat along the measuring chamber, preventing formation of water drops. Even if the above-mentioned solution is effective in preventing water drops formation on optical elements, it worsens the temperature control as larger oscillations in temperature on emitter and receiver stages are introduced. To correctly evaluate the effect of unstable temperature on LED and PD efficiency, preliminary both theoretical and experimental analysis have been carried out, and then a method to compensate the temperature effect has been proposed.

### 2.4. Preliminary Analysis of Temperature Effect on Receiver Stage

**Figure 8:**
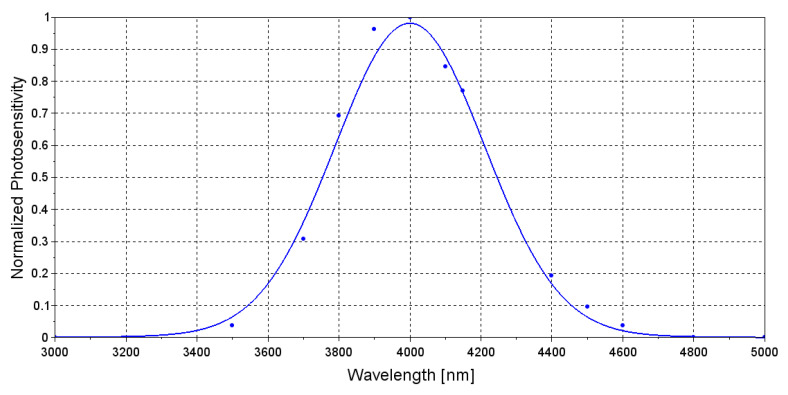
Simulated PD spectral response (solid line) and data provided by the manufacturer (dots) [30].

Temperature increase of the receiver PD determines an increase of both dark current noise and response time. Dark current noise arises from the generation of a current on a photosensitive device even if photons are not detected. The movement of charges that generates the dark current noise mainly depends on the thermal condition of the device, and it increases as the temperature increases. This effect worsens the SNR (signal-to-noise ratio) but can be compensated through postprocessing on the acquired signal. Slower PD response can be compensated using an appropriately long for emission pulse, being sure that the pulse duration allows the PD to reach the steady-state. In our system, the emission pulses are generated at 100 Hz frequency with a pulse duration of 1 ms. To evaluate if the temperature affects the PD photosensitivity (i.e., the ability of the PD to generate current when hit by photons), a theoretical approach can be used. The spectral response of an InAsSb/InAs PD can be described by the sum of two Gaussian curves G1 and G2 [32]:

(3) $$R_{PD}\left(\lambda ,T\right)=R0\cdot [K1\cdot G1\left(\lambda max\left(T\right),\Delta \lambda \left(T\right)\right)+K2\cdot G2\left(\lambda max\left(T\right)-\lambda_{0},\Delta \lambda_{0}\right]$$

where *R*0 is the *PD* integral photodiode sensitivity [A/W], Δλ(*T*) is the FWHM, *λmax*(*T*) is peak wavelength, *K*1,*K*2,λ0 and $\Delta \lambda_{0}$ are adjustable parameters, set in order to fit the spectral response data provided by the manufacturer at *T* = 27 °C (Figure 8). As for the LED modeling: Δλ(*T*) ∼ 0.1 *λmax*(*T*) and d*λmax*/d*T* = 4.5 nm/°C within the temperature range 0 to 50 °C. Solving Equation (3) highlights that increase on *PD* temperature affects its photosensitivity only at high temperatures, whereas the effect on photosensitivity around 40 °C can be considered negligible.

### 2.5. Preliminary Analysis of Temperature Effect on the Emitter Stage

**Figure 9:**
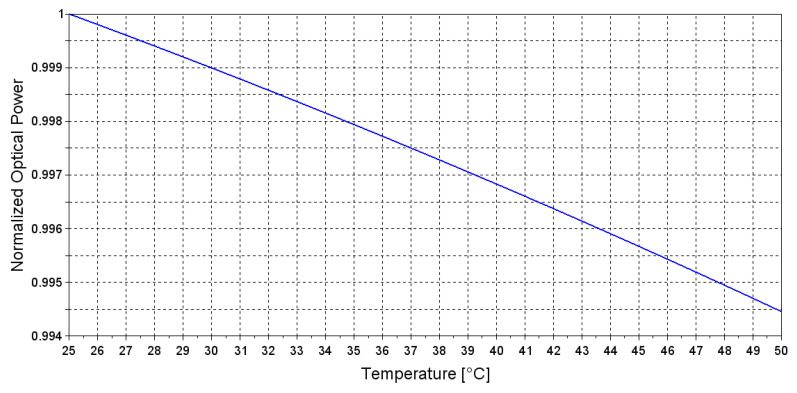
Theoretical evaluation of correlation between emitted optical power and temperature.

The following equation describes the emission of the mid-IR beam by an InAsSb/InAS LED,

(4) $$P_{LED}\left(\lambda ,T\right)=\frac{P0}{\pi }\cdot \frac{\Delta \lambda (T)}{\Delta \lambda {\left(T\right)}^{2}+{\left(\lambda -\lambda max\left(T\right)\right)}^{2}}$$

where *P*0 is the total output power [*μ*W], λ is the wavelength [nm], Δλ and λ*max* are the FWHM (Full Width at Half Maximum) and peak wavelength of the emission spectra, respectively. For λ*max* we considered the value provided by the LED datasheet at 27 °C [30]. Within the temperature range 0 to 50 °C the following relationships are valid, Δλ(*T*) ∼ 0.1 *λmax*(*T*) and d*λmax*/*dT* = 4.5 nm/°C [32]. By solving Equation (4) is possible to describe the mid-IR LED emission spectra at different temperatures. As the temperature increases, the peak wavelength of the emission spectra shifts to higher wavelengths values and at the same time the optical power intensity decreases, as reported in Figure 9 where the total output power of the LED was calculated in the temperature range 25 to 50 °C.

### 2.6. Experimental Analysis

**Figure 10:**
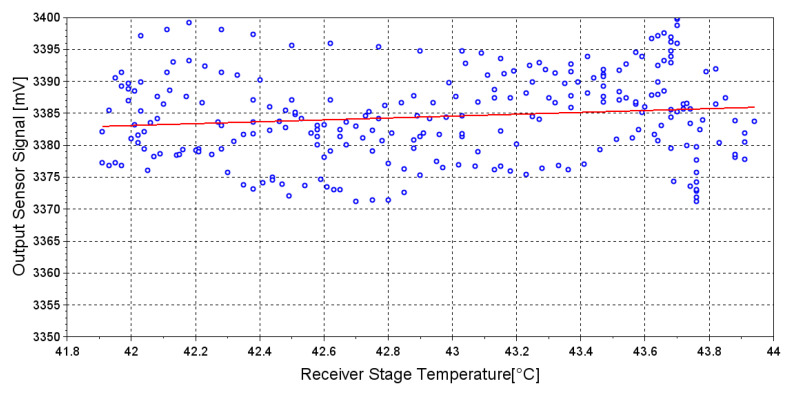
Experimental evaluation of correlation between emitted output power and receiver stage temperature. Blue dots represent the sampled value of the output sensor voltage at several temperatures of the receiver stage. Red line represents the linear regression of the data.

Experimental analysis has been performed to validate the theoretical formulations reported in paragraphs 2.4 and 2.5. To experimentally evaluate the effect of temperature on the receiver element, sensor’s output signal has been recorded activating the heating system only on the receiver stage, in order to avoid interference due to temperature effect on the emitter element. The theoretical formulation expressed by Equation (3) is confirmed experimentally (Figure 10), as we did not observe any correlation between the receiver stage temperature and the output sensor signal (R^2^ = 0.02). Therefore, the PD photosensitivity dependency on temperature is negligible in terms of optical signal variation and does not affect the measurement provided by our sensor.

**Figure 11:**
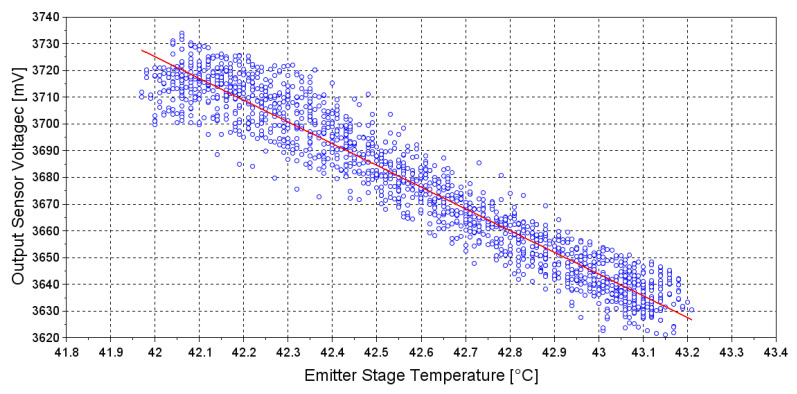
Experimental evaluation of correlation between emitted output power and emitter stage temperature. Blue dots represent the sampled value of the output sensor voltage at several temperatures of the emitter stage. Red line represents the linear regression of the data.

Effect of the temperature on the emitter element has been experimentally studied activating the heating system only on the emitter stage, acquiring the sensor output signal at several temperature values of the emitter stage. The experimental result (Figure 11), shows a negative linear correlation with an R^2^ coefficient of 0.93.

Anyway, even though Figure 9 and Figure 11 both show a negative correlation between emitted optical power and temperature, their slopes are different. In particular, from the theoretical analysis the sensor signal variation due to temperature variation of the emitter element should be negligible, but this is not confirmed experimentally. This is because in theoretical formulation only the contribute of the *LED* is considered, while in the experiment is considered also the contribute of the receiver element. Therefore, to mathematically describe the effect of temperature variation on a single channel mid-IR sensor, both Emitter and Receiver spectral characteristics shall be considered. Equation (5) describes the sensor’s optopair spectral characteristics [22].

(5) $$A\left(\lambda ,\lambda max\left(T\right)\right)=P_{LED}\left(\lambda ,T\right)\cdot R_{PD}\left(\lambda ,T\right)$$

**Figure 12:**
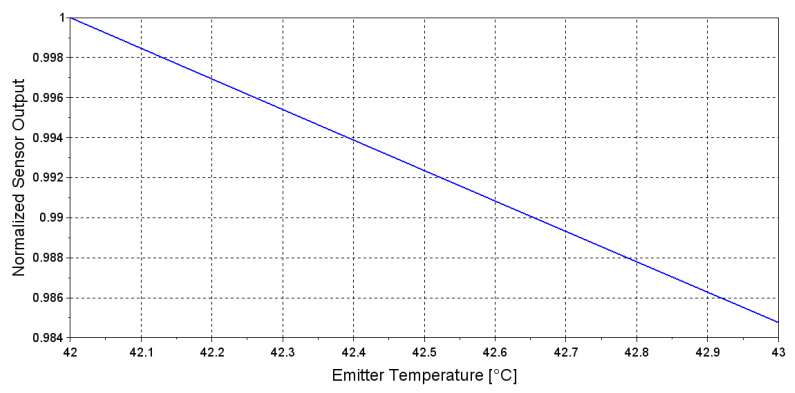
Theoretical evaluation of correlation between sensor’s output and emitter element temperature, using sensor optopair formulation.

Solving Equation (5) for the Emitter temperature range 42 to 43 °C and considering *PD* temperature at 27 °C, allows to simulate the experiment of Figure 11. The result reported in Figure 12 shows that increase of 1 °C reduces the output of 1.5%, while in the experiment of Figure 11 increase of 1 °C reduces the output of 2%. Considering the approximation made by the mathematical model, this result confirm that the theoretical formulation correctly describe the behaviour observed experimentally.

This result confirm that the proposed formulation is suitable for describing the behaviour of a single channel optical sensor.

From the experiment we estimated that the sensor output signal decreases of approximately 70 mV/°C. Considering that in our sensor, the output signal is reduced by approximately 700 mV at maximum measurable value of CO_2_ concentration, and considering the non-linearity of the sensor response described by (2), the above mentioned signal–temperature variability can not be tolerated. Further, a signal decrease due to LED temperature increase was noticed even if the heating system was turned off, due to "self-heating" of the LED. The current circulating in the LED is indeed sufficient to increase the temperature of the optical element, thus affecting its emitted power as discussed before. Therefore, a strategy to avoid the sensor signal variation due to the temperature variation at the emitter stage is necessary. Such strategy to achieve the signal stability is described in detail in the following paragraphs.

### 2.7. Temperature Control Algorithm

The results of the preliminary analysis suggest that temperature variations on the emitter stage represent the major contributor to output sensor signal variation, whereas the temperature effect on the sensitivity of the receiver can be considered negligible. Therefore, a method to compensate the temperature variations on the emitter stage was studied, in order to improve the output signal stability. The proposed method consists of an algorithm for the generation of periodical and controlled temperature variations on the emitter stage, and synchronization of the optical signal acquisition at known temperature conditions. In this way, the optical signal is always sampled at the same thermal conditions, and the signal variations due to heating–cooling dynamics are not taken into account. The developed algorithm is made of two phases:

- an initial phase performed at start-up, necessary to allow the sensor to reach the steady state temperature, and
- a phase in which the heating module is alternatively turned on and off by the CPU.

**Figure 13:**
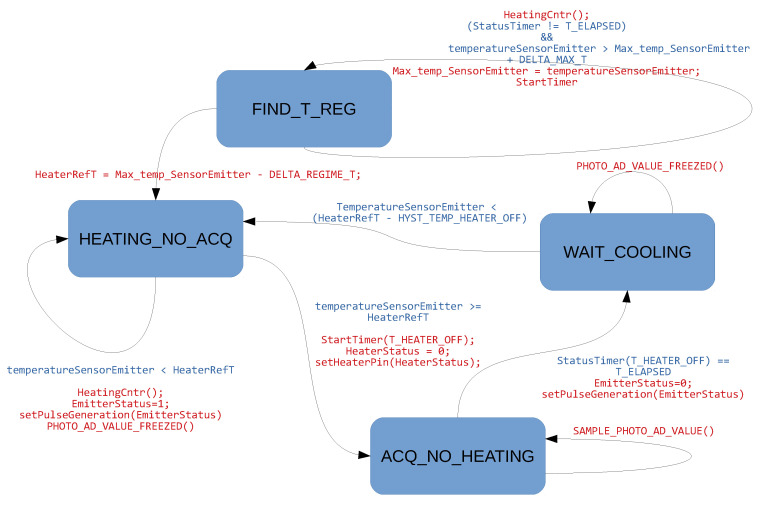
State diagram of Temperature Control Algorithm.

The initial phase (FIND_T_REG state) is necessary to tune the control algorithm on the room temperature and to assure repeatability of the generated temperature oscillations used in the second phase. During the second phase, the CPU generates controlled temperature oscillations switching alternatively on and off the heating system. The heating system is switched off when the emitter stage temperature reaches a threshold value (HeaterRefT) based on the steady state temperature. While the heating system is turned off (ACQ_NO_HEATING state) the output signal is acquired and processed by the CPU through a moving average filter. This condition is maintained for a fixed duration of 45 s. Once the 45 s elapse the algorithm moves to the WAIT_COOLING state in which the signal acquisition is stopped and the sensor output signal is no more updated. During this state the heating system is maintained off until a threshold value is reached (HeaterRefT - HYST_TEMP_HEATER_OFF). Further, in this state, also the emitter LED is turned off in order to remove the LED self-heating due to electrical current circulating. Once reached the threshold value, the algorithm moves to the HEATING_NO_ACQ state in which both the LED and heating system are turned on again. The algorithm then continues cyclically. A schematic representation of the algorithm is reported in Figure 13.

**Figure 14:**
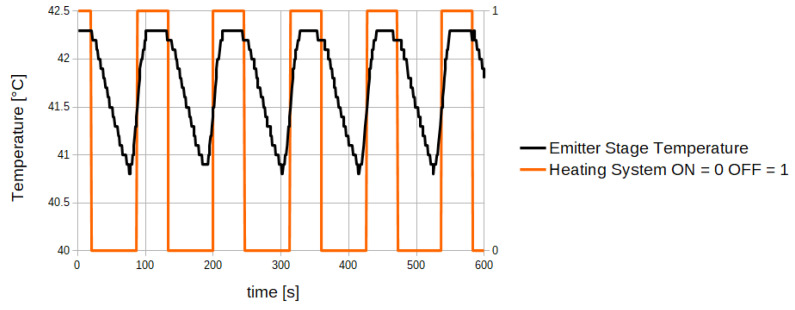
Emitter Stage Temperature trend obtained through the temperature control algorithm.

**Figure 15:**
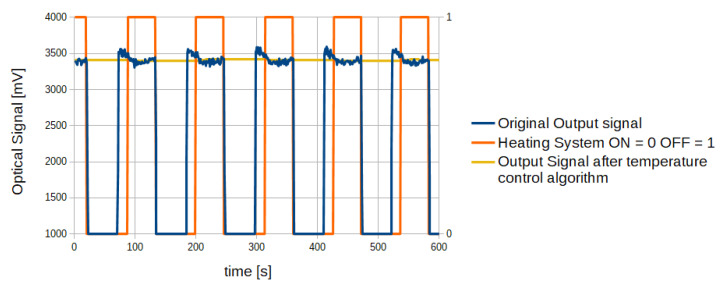
Original output signal (blue) and output signal (yellow) obtained through the temperature control algorithm.

Through the proposed algorithm, the sensor output signal is updated only at the same thermal conditions, therefore the output changes due to temperature variation are avoided. In Figure 14 the emitter stage temperature trend over time with the proposed control algorithm is reported, while Figure 15 reports the sensor output signal obtained from the algorithm.

As shown in Figure 14, the emitter stage temperature reaches the same values at each acquisition cycle (HEATING_NO_ACQ state). Therefore, as reported in Figure 15, the output signal obtained through averaging operation of the signal acquired during the HEATING_NO_ACQ state is much more stable than the original signal. Without the implementation of the proposed algorithm, temperature dynamics on the emitter stage is characterized by oscillations without a defined period. Therefore, the use of simple filtering methods such as a moving average on the acquired signal result poorly effective as the temperature–signal dependency remains.

## 3. Results

### 3.1. Sensor Validation in Experimental Laboratory Setting

**Figure 16:**
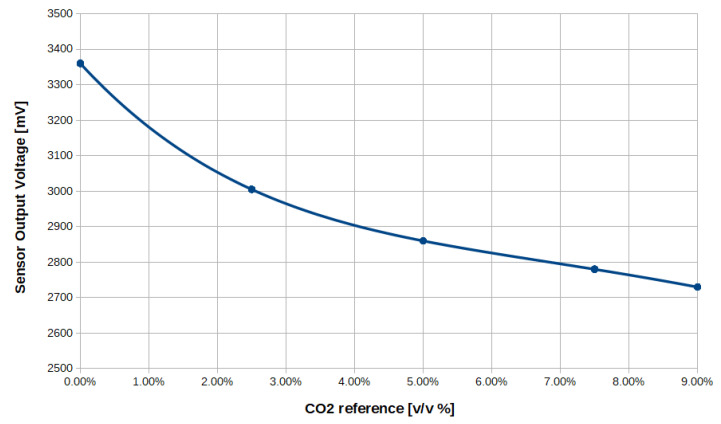
Example of calibration curve. Dots represent the values used for polynomial interpolation.

The developed CO_2_ sensor and temperature control algorithm was tested in terms of CO_2_ measurement accuracy. Before testing, the sensor was calibrated by using gas tanks containing gas mixtures of air and CO_2_ with known concentrations. The measurement range for CO_2_ concentration was 0–9%, as the developed sensor is intended to be used for treating patients in hypercapnic conditions (i.e., P_a_CO_2_ higher than 50 mmHg [33], corresponding to a CO_2_ concentration of approximately 6.5%). The gas was let flow through the sensor and the output signal was recorded through a PC connected to the sensor via RS-485 communication line. The following CO_2_ concentrations were used for the calibration procedure; 0%, 2.5%, 5%, 7.5%, and 9%. The collected data were used to obtain a calibration curve through polynomial interpolation (Figure 16).

**Figure 17:**
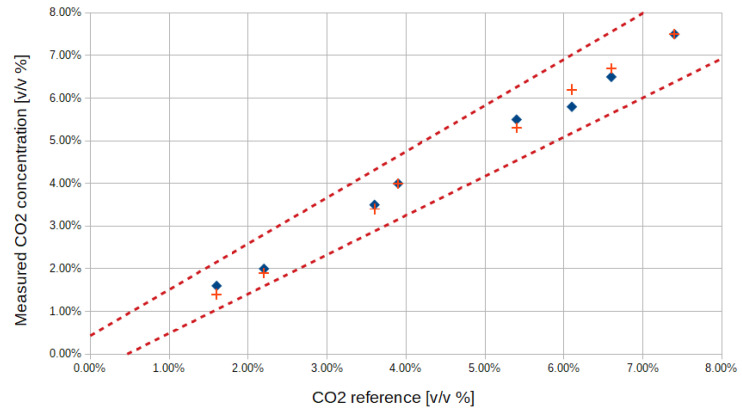
CO_2_ concentration measurement provided by the newly developed sensor (blue diamonds) and by the “gold standard” device (orange crosses). Dashed lines represent the ISO 80601-2-55 error limits.

The parameters that define the calibration curve were then loaded in the CO_2_ sensors. Sensor accuracy was verified by comparing its measurement with the ones obtained by a “gold standard” device (Medtronic Microcap Plus Capnograph). The accuracy of the two sensors was compared using a mixture of air and CO_2_ at several concentrations. In order to control the CO_2_ concentration of the gas mixture used for the test, it was analyzed through a bench gas analyser (Servomex MiniMP 5200). Test results are reported in Figure 17.

From data reported in Table 1, the average absolute error obtained for the newly developed sensor (0.13%) is similar to the one obtained by the reference device (0.15%), that is already validated for clinical practice. Further, the accuracy level shown by the new sensor complies with requirements prescribed by ISO 80601-2-55. These results demonstrate the efficiency of the temperature control algorithm in terms of output signal stability, therefore the reliability of the newly developed sensor.

### 3.2. In Vivo Sensor Validation

To validate the new device in vivo, we tested the sensor in an animal model, and compared the obtained measurements with the ones taken with Medtronic Microcap Plus Capnograph. Data were collected during the execution of experiments described in [7], in which pigs with induced cardiogenic shock were undergoing ECMO procedure. Measurements from the newly developed capnometer and the reference device were taken at the MO exhaust port. Further, the *GF* measurement taken at the inlet port of the MO through our flow sensor and the one provided by a reference device were compared. Finally, *VCO*_2_ data obtained by *CO*_2_ measurement from our system were evaluated against *VCO*_2_ calculated through data gathered by the reference device. Both for the newly developed sensor and reference device the following formula was used for determination of *VCO*_2_,

(6) $$VCO_{2}\left(mL/min\right)=\frac{GF\left(mL/min\right)\cdot CO_{2}\%}{100}$$

As during in vivo tests the *CO*_2_ concentration cannot be set, to validate our sensor we focused on the error obtained comparing the measurements from our sensor with the ones taken with the reference device, that is routinely used in clinical practice. Results are reported in Table 2.

These results show that the newly developed CO_2_ sensing system represents a reliable solution in the clinical environment. Based on the results obtained both in vitro and in clinical environment, the developed sensor has obtained the CE mark and it is currently used in intensive care units connected to multiparametric monitoring systems for ECMO procedures and to ECCO2R devices.

## 4. Discussion and Conclusions

In this paper, we present a new sensor specifically designed for measurement of carbon dioxide concentration in the exhaust gas of a membrane oxygenator during extracorporeal procedures. The developed sensor is made up by two sections: one for measurement of the gas flow applied to a membrane oxygenator, and the other for the measurement of carbon dioxide concentration in the exhaust gas, which represents the CO_2_ removed from the patient’s blood. The proposed CO_2_ sensor is designed as a Main Stream sensor with a single channel optical architecture, avoiding use of optical filters and therefore obtaining a simple, cheap and easy to assemble system. As the oxygenator exhaust gas is characterized by an high amount of water vapor, condensation at the exhaust connector is easy to occur. Therefore, in order to avoid water vapor condensation within the optical sensor, with consequent degradation of the optical signal, a heating module is implemented in the CO_2_ measurement section. Even though the implemented heating system prevents water vapor condensation, temperature variations affect the performance of the optical elements. Temperature effect on the optical elements used in the CO_2_ sensor was analyzed both theoretically and experimentally, highlighting the strong correlation between emitter LED temperature and acquired optical signal. Even though both theoretical evaluation and experimental results highlight a negative correlation between emitted optical power and emitter stage temperature, reported in Figure 8 and Figure 11, respectively, the two relationship are characterized by different slopes. This result can be explained considering that, in the experimental setup, the sensor photodiode contributes to the variation of sensor’s output, while in Equation (4) this contribute is not taken into account. To correctly describe the experimental setup, the formulation reported in Equation (5) has been used. This formulation considers both the emitter and receiver contribute to the sensor output. Using it to simulate the experimental setup the obtained relationship between sensor output and emitter temperature (shown in Figure 12) is close to the one obtained experimentally (Figure 11). The mathematical model used was confirmed valid and useful to evaluate the effect of temperature changes introduced by the heating system of our device on the optical elements. Further, as reported in [32], the mathematical modeling can be also used to evaluate sensor sensitivity. Therefore, the adopted mathematical model represents the basis for further improvement of our device since allows to explore the behavior of our sensor for different conditions of use, e.g., higher temperatures, different optical path length, and higher CO_2_ concentration. In order to remove the signal–temperature dependency and therefore improve sensor sensitivity, an algorithm for heating system control was implemented. The proposed solution consists in the generation of controlled and repeatable temperature oscillations and acquisition of the sensor’s output signal always at the same thermal conditions. Use of the proposed temperature control algorithm, results in a stable output signal since only samples acquired at the same thermal conditions are considered. The main drawback of the implemented solution is the low time resolution of the measurement. Considering the duration of a thermal cycle, there is a 90 seconds delay in the detection of a CO_2_ concentration variation. Anyway, we do not consider this a major problem considering that the sensor is intended for long term therapy that lasts for days or weeks, meaning that the data gathered through the sensors are used to get long-term information about therapy progress and trends. Accuracy of the developed sensor has been compared against a CO_2_ sensor used in clinical practice both through laboratory test and in vivo test. The results suggest that the developed system allows a reliable measurement of gas flow and carbon dioxide concentration, and therefore of carbon dioxide removal rate VCO_2_. Is important to highlight that, the reference device considered as gold standard in our experiments is a CO_2_ sensor intended for respiratory monitoring, and it is able to perform the measurements only in "intermittent" gas flow conditions like breathing. Therefore, during our experiments the reference device was manually connected/disconnected from the MO exhaust port. This means that sensors designed for respiratory monitoring, like the one used as gold standard in our experiments, are not suitable for use in ECLS procedure due the impossibility of measurement in continuous gas flow conditions. A far as the author knows, only another device for measuring of CO_2_ concentration specifically designed for ECLS application is available at the moment, the CO_2_ sensor of Spectrum M4 system (Spectrum Medical, Gloucester, England), but no information about its functioning or its performances in terms of CO_2_ concentration accuracy was found nor it was possible to compare this device with our sensor experimentally. The developed sensor fulfills the requirements prescribed by ISO 80601-2-55 standard relevant to the accuracy of measurements of carbon dioxide concentration and has already obtained the CE mark. It is currently in use in intensive care units connected to multi-parametric monitoring systems for ECMO procedures and to ECCO_2_R devices. From the feedback obtained through clinical practice, a more deep validation will be performed.

## Figures and Tables

**Table 1:** Comparison between measurements get by the developed sensor and a “gold standard” device for several CO_2_ concentration values. Error is expressed as average value and standard deviation (SD).

CO_2_ Concentration Set-Point [*v*/*v* %]	Developed CO_2_ Sensor	“Gold Standard” Sensor
1.60%	1.60%	1.40%
2.20%	2.00%	1.90%
3.60%	3.50%	3.40%
3.90%	4.00%	4.00%
5.40%	5.50%	5.30%
6.10%	5.80%	6.20%
6.60%	6.50%	6.70%
7.40%	7.50%	7.50%
Average Error ± SD	0.13 ± 0.09%	0.15 ± 0.07%

**Table 2:** Error between measurement taken with the newly developed system and the “gold standard” devices for CO_2_ concentration, Gas Flow, and VCO_2_. Error is expressed as average value and standard deviation (SD).

Gas Flow	Gas Flow	CO_2_ Concentration	CO_2_ Concentration	VCO_2_	VCO_2_
Developed Sensor	“Gold Standard”	Developed Sensor	“Gold Standard”	Developed Sensor	“Gold Standard”
[L/min]	Sensor [L/min]	[*v*/*v* %]	Sensor [*v*/*v* %]	[mL/min]	Sensor [mL/min]
1.60	1.50	3.80%	3.80%	60.8	57.0
1.00	1.00	7.00%	6.90%	70.0	69.0
1.00	1.00	7.10%	7.00%	71.0	70.0
1.10	1.00	8.20%	8.30%	90.2	83.0
1.50	1.40	7.60%	7.60%	114.0	106.4
2.20	2.10	5.20%	5.00%	114.4	105.0
3.60	3.60	3.60%	3.40%	129.6	122.4
0.80	0.70	5.00%	4.80%	40.0	33.6
3.10	3.00	4.40%	4.40%	136.4	132.0
4.50	4.50	3.60%	3.40%	162.0	153.0
Gas Flow Average Error ± SD [L/min]		CO_2_ Concentration Average Error ± SD [*v*/*v*%]		VCO_2_ Average Error ± SD [mL/min]	
0.06 ± 0.05		0.11 ± 0.09		5.7 ± 3	
